# Cost-effectiveness of colon capsule endoscopy in colorectal cancer screening: a modeling study

**DOI:** 10.1055/a-2658-0960

**Published:** 2025-08-01

**Authors:** Lucie de Jonge, Esther Toes-Zoutendijk, Rosita van den Puttelaar, Fanny E. R. Vuik, Manon C. W. Spaander, Iris Lansdorp-Vogelaar

**Affiliations:** 1 Department of Public Health, Erasmus MC University Medical Center, Rotterdam, Netherlands; 2 Department of Gastroenterology and Hepatology, Erasmus MC University Medical Center, Rotterdam, Netherlands

## Abstract

**Background:**

The most used primary colorectal cancer (CRC) screening tests are the fecal immunochemical test (FIT) and colonoscopy. However, colonoscopy is an invasive procedure with possible (fatal) complications and FIT has shortcomings in test sensitivity. Colon capsule endoscopy (CCE) could be an alternative, but long-term effects are unknown. We assessed the cost-effectiveness of CCE in CRC screening.

**Methods:**

We simulated a Dutch cohort born between 1938 and 1957 for eight strategies: biennial FIT screening with cutoffs of 15 (FIT15) and 47 (FIT47) micrograms of hemoglobin per gram feces (µg Hb/g); biennial and triennial CCE screening; CCE after a FIT-negative result of 15–47 µg Hb/g (CCE triage); CCE after positive FIT using 15 and 47 µg Hb/g cutoffs; and 10-yearly colonoscopy screening. Three adherence scenarios were considered. We estimated lifetime CRC incidence, mortality, life years gained, and number of tests required. A cost-effectiveness analysis was performed to determine cost-effectiveness of each strategy.

**Results:**

Triennial CCE and CCE triage screening had similar long-term outcomes to biennial FIT47. At 100% adherence, biennial CCE screening reduced CRC incidence from 79 to 49 cases (38.0% reduction) and mortality from 36 to 17 deaths (52.8% reduction) per 1000 individuals versus no screening. Life years gained increased to 155 per 1000 individuals versus 115 with biennial FIT47 (34.8% increase). However, these increases came at high financial cost, and CCE cost-effectiveness was dominated by biennial FIT and 10-yearly colonoscopy.

**Conclusion:**

CCE was not cost effective for CRC screening compared with biennial FIT and 10-yearly colonoscopy.

## Grapical abstract

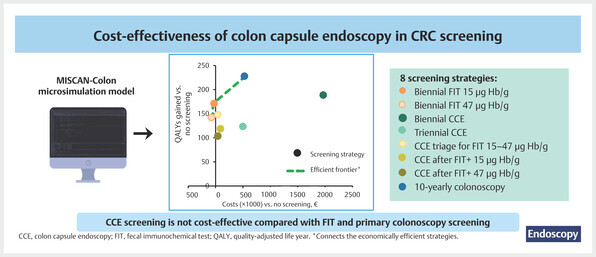

## Introduction

The most used primary colorectal cancer (CRC) screening tests are colonoscopy and the fecal immunochemical test (FIT). The FIT is a stool-based test to detect hemoglobin in the stool. Participants need to sample their stool and return it to the laboratory. If the hemoglobin concentration is above a prespecified level, participants are referred for follow-up colonoscopy. A colonoscopy is an endoscopic examination of the large bowel by a camera attached to a flexible tube. Colonoscopy has a high sensitivity and specificity but is an invasive procedure that can cause (fatal) complications and requires considerable endoscopy capacity if used for primary screening purposes. Although FIT is relatively user-friendly with minimal patient burden, it has its shortcomings in test sensitivity.

Other screening tests such as computed tomography colonography (CTC) and colon capsule endoscopy (CCE) could be alternatives. CTC is an imaging examination of the colon and is minimally invasive and already used in some CRC screening programs as an alternative to colonoscopy. CCE requires the patient to swallow a capsule that has cameras at both ends 1 . It takes 35 images per second as it passes through the digestive tract. The images are automatically recorded and sent to a sensor. This sensor needs to be worn around the body, for example around the abdomen, and information is saved using a larger recording device, which can be worn as a bag. All full recordings give a complete overview of the digestive tract.

Although CCE requires bowel preparation as for colonoscopy, it has several advantages over colonoscopy: it is less invasive, has minimal complication risks, does not require sedation, and can be used at home. Moreover, individuals who need to undergo a colonoscopy or CTC, often prefer to undergo a CCE 2 . Finally, CCE has shown promising results, with a sensitivity for polyps ≥10 mm ranging from 84% to 97%, and specificity ranging from 66% to 97% 2 . Moreover, CCE has shown superior results in the detection of polyps ≥6 mm compared with CTC 3 . The Population cOlon cancer screening by Capsule endoscopy (ORCA) study was initiated to investigate the population-based prevalence of gastrointestinal abnormalities at CCE in asymptomatic individuals aged 50–75 years. Data from this study were used in the current study for the age distribution, costs of CCE, and participation rate 4 .

In addition to CCE being a potential suitable alternative in primary screening, it can also be used in diagnostic follow-up and as a triage test. It has been shown that individuals with hemoglobin concentrations just below the cutoff in previous screening are at higher risk of having future advanced neoplasia or CRC 5 . However, these individuals will not be referred for a follow-up colonoscopy. Therefore, those individuals may benefit from additional screening with CCE with higher accuracy than FIT. Given the lack of literature on the long-term effectiveness and cost-effectiveness of primary CCE screening or CCE as a triage test, the aim of this study was to evaluate the long-term cost-effectiveness of CCE screening and/or triage in comparison with established CRC screening strategies.

## Methods

We used the MIcrosimulation Screening ANalysis model for CRC (MISCAN-Colon) to simulate a population cohort that was invited to primary CCE screening, similarly to the ORCA study 4 . We assessed eight different screening strategies to determine long-term CRC screening outcomes and performed a cost-effectiveness analysis from a healthcare sector perspective.

### MISCAN-Colon

The MISCAN-Colon model is a well-established and validated microsimulation model developed by the Department of Public Health at the Erasmus Medical Center (Rotterdam, the Netherlands). The model has been described previously in the literature 6 7 . In brief, the model simulates the life histories of a large population of individuals from birth to death. In addition, the model simulates the development of CRC through the adenoma–carcinoma sequence. As each simulated individual ages, one or more adenomas may develop, and these adenomas can progress in size from small (≤5 mm) to medium (6–9 mm) to large (≥10 mm). Some adenomas can develop into preclinical cancer, which may progress through cancer stages I to IV. At any time during the development of the disease, symptoms may present, and CRC may be diagnosed. By introducing screening, the simulated life histories may be altered through the detection and removal of adenomas or CRC at an earlier stage with a more favorable prognosis. By comparing the life histories of a simulated population undergoing screening with the corresponding life histories in a simulated population without screening, MISCAN-Colon can quantify the effectiveness and costs of screening.

MISCAN-Colon was adjusted to match age-specific CRC incidence in the Netherlands before the introduction of screening in 2014 by calibrating to data on age-, stage- and location-specific CRC incidence between 2009 and 2013 obtained from the Netherlands Cancer Registry and to age-specific prevalence and multiplicity distribution of adenomas from autopsy and colonoscopy 8 9 10 11 12 13 14 15 16 17 18 . FIT characteristics in MISCAN-Colon were adjusted so that the simulated positivity rate and detection rates were similar to those observed in the Dutch CRC screening program. Test characteristics of the second-generation CCE capsule have been previously calibrated in MISCAN-Colon by Peterse et al. 19 using findings of Rex et al. 20 . Input for the reach of the test in the model was obtained from data in the ORCA trial 21 . Test characteristics of colonoscopy were obtained from published literature 22 . An overview of all test characteristics can be found in Table 1 .

**Table:** **Table 1** Characteristics of colorectal cancer screening modalities.

	FIT15	FIT47	CCE	Colonoscopy
Test characteristics, %				
Sensitivity				
Small adenoma (≤5 mm) ^1^	0	0	0	75.0
Medium adenoma (6–9 mm)	48.1	19.3	86.5	85.0
Large adenoma (≥10 mm)	55.0	36.8	87.5	95.0
CRC early preclinical ^2^	34.3	28.8	87.5	95.0
CRC late preclinical ^2^	70.8	65.2	87.5	95.0
Specificity	96.3	98.9	83.0	100
Perforation and associated complications (e.g. infection)				0.00001
Reach ^3,4^				
Rectum			55.8	100
Rectosigmoid			–	–
Sigmoid			–	–
Descending colon			94.2	–
Transverse colon			95.4	–
Ascending colon			97.8	–
Cecum			99.3	96.0
CCE, colon capsule endoscopy; CRC, colorectal cancer; FIT, fecal immunochemical test; FIT15, FIT using a positivity cutoff of 15 µg Hb/g feces; FIT47, FIT using a positivity cutoff of 47 µg Hb/g feces; ORCA, Population cOlon cancer screening by Capsule endoscopy. ^1^ Sensitivity for small adenomas was assumed to be zero. ^2^ It was assumed that the probability of a CRC bleeding, and thus the sensitivity of a FIT for CRC, depends on the time until clinical diagnosis 23 . ^3^ Input for reach of CCE was obtained from data from the ORCA trial 21 . ^4^ The probability of a test reaching a location without a prespecified probability is defined by a piecewise linear function using the next locations with a prespecified probability.				

### Screening strategies

**Fig. 1:**
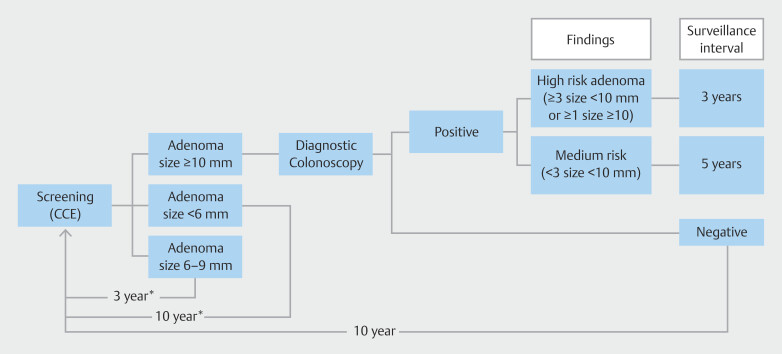
Screening pathway used in the MISCAN-Colon microsimulation model for the colon capsule endoscopy 16 . *These intervals are adjusted to 2 years in biennial CCE screening strategy. CCE, colon capsule endoscopy.

We simulated a Dutch cohort of 100 million individuals from birth to death (restricted at age 100) born between 1938 and 1957. Eight different screening strategies were simulated: 1) biennial FIT screening using a positivity cutoff of 47 micrograms of hemoglobin per gram feces (µg Hb/g) (FIT47); 2) biennial FIT screening using a positivity cutoff of 15 µg Hb/g feces (FIT15); 3) biennial CCE screening; 4) triennial CCE screening; 5) CCE triage screening; 6) CCE screening after positive FIT15; 7) CCE screening after positive FIT47; and 8) 10-yearly colonoscopy screening. In all strategies, individuals were invited according to the current age range used in the Dutch CRC screening program, which is from age 55 to 75 years. In the first two strategies, we simulated the Dutch CRC screening strategy with FIT as the primary screening test with two different positivity cutoff values. In the third and fourth strategies, we replaced FIT with biennial and triennial CCE, respectively, as the primary screening test, with follow-up CTC according to guidelines 16 ( Fig. 1 ). So, individuals with a medium-risk adenoma are reinvited for a CCE after 2 years in biennial CCE and after 3 years in triennial CCE. In the fifth strategy, CCE triage screening, CCE was used as a triage test for those with a FIT-negative result between 15 and 47 µg Hb/g feces (see **Fig. 1s** in the online-only Supplementary material). Next, in the sixth and seventh strategies, individuals with a positive FIT using a positivity cutoff of 15 (FIT15+) or 47 (FIT47+) µg Hb/g feces, respectively, were referred for a follow-up CCE. Finally, a 10-yearly primary colonoscopy screening was simulated.

We assumed three adherence scenarios to all screening tests:

1. 100% to all screening tests
2. 25% in colonoscopy, biennial, and triennial CCE screening 4 , and 75% in biennial FIT screening 24
3. 25% in colonoscopy screening, 50% in biennial and triennial CCE screening, and 75% in biennial FIT screening.

In all three scenarios, we assumed 100% adherence to diagnostic and surveillance tests.

### Outcomes

For all strategies, the model estimated the number of FITs, CCEs, and colonoscopies, and the number of complications. Long-term screening outcomes were CRC incidence, CRC-related mortality, number of life years, life years gained compared with no screening, number of quality-adjusted life years (QALYs), and QALYs gained compared with no screening. QALY is a measure of length of life and quality of life, determined by correcting the life years with losses in quality of life. Factors used to correct life years for quality of life are called utilities ( Table 2 ). Lifetime costs associated with screening and CRC treatment were estimated ( Table 2 ). The costs of FIT (FIT kits, postage of kits and stool samples, and analysis) used in the screening program were derived from the Dutch National Institute for Public Health and Environment. Costs for colonoscopy, polypectomy, and complications from colonoscopy, as well as costs for cancer care were based on retrospective chart reviews. We estimated the average utilization of healthcare products by patients with CRC within the Diagnosis and Treatment Combinations (DTC) system in the Netherlands. This was then multiplied by the average price of all hospitals in the Netherlands for these services based on the reimbursement 25 . Costs for CRC treatment were divided into three clinically relevant phases of care: initial, continuous, and terminal. Initial care costs were based on DTC rates, except for oxaliplatin. The costs for oxaliplatin were derived from the Dutch Health Care Insurance Board. We assumed that during continuous care, individuals would follow the Dutch CRC treatment guidelines and costs for periodic control were based on DTC rates. Terminal care costs were based on a Dutch last-year-of-life cost analysis 26 . We assumed that these costs increased with stage at diagnosis, at a rate observed for US patients 27 . Dutch terminal care costs for individuals who died of CRC were approximately 40% of the US costs. We therefore assumed that terminal care costs for patients with CRC who die of other causes were also 40% of the US costs.

**Table:** **Table 2** Screening costs and utility losses associated with colorectal cancer screening and treatment.

	Costs, € (2018)	Probabilistic sensitivity analysis, gamma distribution ^1^ , 95%CI	Utility losses
Per FIT	22	11.00–44.00	Positive: 0.00133 Negative: 0.000063
Per CCE	600	300–1200	Cancer/adenoma: 0.001692 Negative: 0.000425
Per colonoscopy	686	343–1372	0.0055
Per polypectomy	295	147.50–590	
Per perforation during colonoscopy	2735	9880.52–39 522.08	0.0384
Treatment per life year with cancer care ^2^			
Initial year			
Stage I CRC	22 859	11 429.50–45 718	0.12
Stage II CRC	19 415	9707.50–38 830	0.18
Stage III CRC	36 633	18 316.50–73 266	0.24
Stage IV CRC	35 513	17 756.50–71 026	0.7
Ongoing			
Stage I–IV CRC	483	241.50–966	I: 0.05; II: 0.05; III: 0.24; IV: 0.7
Terminal year, dying of CRC 27			
Stage I CRC	24 859	12 429.50–49 718	0.7
Stage II CRC	24 859	12 429.50–49 718	0.7
Stage III CRC	26 280	13 140–52 560	0.7
Stage IV CRC	35 513	17 756.50–71 026	0.7
Terminal year, dying of other causes 27			
Stage I CRC	6250	3125–12 500	0.05
Stage II CRC	5682	2841–11 364	0.05
Stage III CRC	7387	3693.50–14 774	0.24
Stage IV CRC	19 887	9943.50–39 774	0.7
CCE, colon capsule endoscopy; CRC, colorectal cancer; DTC, Diagnosis and TreatmentCombinations; FIT, fecal immunochemical test; ORCA, Population colon cancer screening by Capsule endoscopy. ^1^ 95%CIs were derived by halving and doubling the mean value. The mean and SD, μ and σ, respectively, were derived to obtain shape parameter k and scale parameter θ using method of moments by $k=\frac{\mu^{2}}{\sigma^{2}}\;$ and $\theta =\frac{\sigma^{2}}{\mu }$ ^2^ Costs for CRC treatment were divided into three clinically relevant phases of care: initial, continuous, and terminal. Initial care costs were based on DTC rates, except for oxaliplatin. The costs for oxaliplatin were derived from the Dutch Health Care Insurance Board. We assumed that during continuous care, individuals would follow the Dutch CRC treatment guidelines and costs for periodic control were based on DTC rates. Terminal care costs were based on a Dutch last-year-of-life cost analysis 27 . We assumed that these costs increased with stage at diagnosis, at a rate observed for US patients 28 . Dutch terminal care costs for individuals who died of CRC were approximately 40% of the US costs. We therefore assumed that terminal care costs of patients with CRC who die of other causes were also 40% of the US costs.			

### Cost-effectiveness analysis

In the cost-effectiveness analysis, strategies were ranked according to their costs and plotted in a cost-effectiveness plane. Strategies that cost more than (a combination of) other strategies while gaining fewer QALYs were considered inefficient and therefore dominated. For the remaining strategies, cost-effectiveness was expressed by the incremental cost-effectiveness ratio (ICER) as incremental cost per QALY gained compared with the next less-effective strategy. These strategies were connected in the cost-effectiveness plane, which is called the efficient frontier. The willingness-to-pay (WTP) threshold was set at €20 000 per QALY gained. The strategy with the highest ICER below the WTP threshold was considered the most efficient strategy.

### Sensitivity analysis

We conducted four sensitivity analyses to assess the robustness of our results. First, we varied the intervals in the surveillance scheme for CCE and colonoscopy. The interval after a negative CCE or colonoscopy was adjusted to 5 years instead of 10 years, and the interval after detection of medium-risk adenomas at colonoscopy was adjusted to 3 years instead of 5 years. Second, we decreased participation in primary screening to 25%, 50%, and 75%. Third, the CCE recording needs to be reviewed by an endoscopy nurse, clinical nurse specialist, or nurse, which requires staff hours and thereby costs. The reviewing time takes on average 55 minutes 28 and, based on the ORCA study (data unpublished), we have assumed a unit cost per CCE for reviewing of €150. Finally, we performed a threshold analysis for the unit costs of CCE to find the point at which CCE screening becomes cost-effective.

### Probabilistic sensitivity analysis

In the probabilistic sensitivity analysis, we assessed the uncertainty around all costs associated with screening and CRC treatment to evaluate future economic improvements and changes in healthcare costs. For every strategy, we performed 1000 simulations assuming 100% adherence each containing a different set of costs drawn from the gamma probability distribution ( Table 2 ). We chose this distribution because it is well equipped to generate a distribution for non-negative numbers ( Table 2 ).

## Results

### Base case

Without screening, lifetime CRC incidence and mortality would be 79 and 36 per 1000 simulated individuals, respectively ( Table 3 ). Introducing screening between the ages of 55 and 75 reduced both CRC incidence and mortality in all eight screening strategies for all three adherence assumptions. At 100% adherence, FIT47 reduced CRC incidence and mortality to 53 cases (32.9% reduction) and 21 deaths (41.7% reduction) per 1000 individuals, and FIT15 reduced CRC incidence and mortality to 46 cases (41.8% reduction) and 18 deaths (50.0% reduction) per 1000 individuals, respectively. Biennial CCE screening reduced CRC incidence and mortality to 49 cases (38.0% reduction) and 17 deaths (52.8% reduction) per 1000 individuals, respectively. Triennial CCE, CCE triage, and CCE after FIT15+ and FIT47+ showed similar CRC incidence and mortality as FIT47. Finally, 10-yearly colonoscopy screening resulted in the largest reduction, with a CRC incidence and mortality of 29 cases (63.3% reduction) and 11 deaths (69.4% reduction) per 1000 individuals, respectively. At imperfect adherence assumptions, the reductions were smaller, especially for CCE and colonoscopy screening at 25% adherence.

**Table:** **Table 3** Lifetime modeling outcomes (discounted at 3%) per 1000 simulated Dutch individuals for a situation without screening, fecal immunochemical test screening, colon capsule endoscopy (CCE) screening, CCE triage screening, and colonoscopy screening for three adherence scenarios.

	FITs	CCEs	Colonoscopies	Complications	CRC cases	CRC deaths
No screening	–	–	–	–	79	36
100% adherence						
Biennial FIT47	8659	–	516	0.07	53	21
Biennial FIT15	7875	–	829	0.09	46	18
CCE triage	8124	231	541	0.08	53	21
CCE after FIT47+	8895	253	312	0.08	62	25
CCE after FIT15+	8296	468	362	0.07	59	24
Triennial CCE	–	1939	423	0.09	59	24
10-yearly colonoscopy	–	–	3089	0.07	29	11
Biennial CCE	–	7459	530	0.11	49	17
75% adherence to FIT screening, CCE triage screening, and after FIT+; 25% adherence to CCE and colonoscopy screening						
Biennial FIT15	6120	–	710	0.08	49	20
Biennial FIT47	6608	–	443	0.07	56	23
CCE triage	6285	186	474	0.08	55	22
CCE after FIT47+	6763	209	277	0.06	64	27
CCE after FIT15+	6401	383	330	0.07	61	25
10-yearly colonoscopy	–	–	918	0.04	58	25
Triennial CCE	–	911	232	0.06	68	30
Biennial CCE	–	1925	320	0.07	63	26
75% adherence to FIT screening, CCE triage screening, and after FIT+; 50% adherence to CCE screening; 25% adherence to colonoscopy screening						
Biennial FIT15	6120	–	710	0.08	49	20
Biennial FIT47	6608	–	443	0.07	56	23
CCE triage	6285	186	474	0.08	55	22
CCE after FIT47+	6763	209	277	0.06	64	27
CCE after FIT15+	6401	383	330	0.07	61	25
10-yearly colonoscopy	–	–	918	0.04	58	25
Triennial CCE	–	1419	336	0.07	63	27
Biennial CCE	–	3783	439	0.10	56	22
CCE, colon capsule endoscopy; CRC, colorectal cancer; FIT, fecal immunochemical test; µg Hb/g, microgram hemoglobin per gram feces; FIT15, FIT using a positivity cutoff of 15 µg Hb/g feces; FIT47, FIT using a positivity cutoff of 47 µg Hb/g feces; FIT+, positive FIT; FIT15+, positive FIT15; FIT47+, positive FIT47; QALY, quality-adjusted life year; ICER, incremental cost-effectiveness ratio.						

At 100% adherence, the number of FITs used in the biennial FIT15 screening and CCE triage screening were similar (7875 FITs in FIT15 and 8124 in CCE triage). Strategy FIT47 required a slightly higher number of FITs (8659). In the CCE triage screening, 231 CCEs were required in addition to the 8124 FITs. This was similar for CCE after FIT47+. Triennial CCE screening resulted in a much lower number of CCEs required compared with biennial CCE screening (1939 vs. 7459 per 1000 individuals). Colonoscopy demand ranged from 312 to 3089 per 1000 individuals, with the lowest demand required for CCE after FIT47+ and the highest for 10-yearly colonoscopy screening. The number of tests required was lower with reduced adherence.

### Costs and cost-effectiveness analysis

**Fig. 2:**
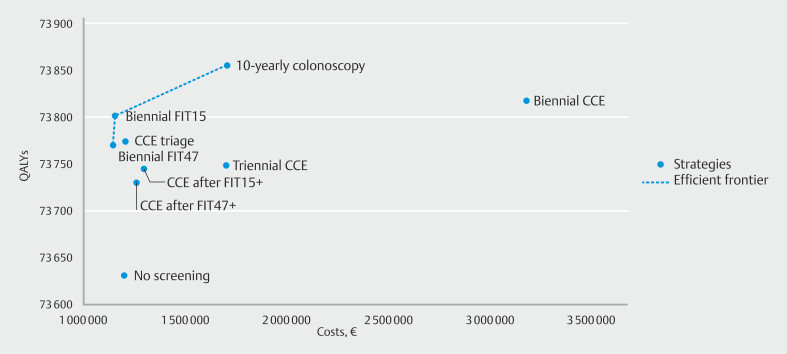
Lifetime costs and life years (discounted at 3%) per 1000 simulated Dutch individuals of all colorectal cancer screening strategies at 100% participation rate and a strategy without screening, with the efficient frontier connecting the economically efficient strategies. CCE, colon capsule endoscopy; FIT, fecal immunochemical test; FIT15, FIT using a positivity cutoff of 15 µg Hb/g feces; FIT47, FIT using a positivity cutoff of 47 µg Hb/g feces; QALY, quality-adjusted life year.

Without screening, the total cost of CRC care was estimated to be €1 200 737 per 1000 individuals ( Table 4 ). At 100% adherence, introducing screening with biennial FIT decreased costs to up to €1 154 074 per 1000 individuals, depending on the cutoff used. The highest costs were estimated for biennial CCE screening (€3 178 861 per 1000 individuals). The number of life years gained compared with no screening ranged from 83 to 187 per 1000 individuals. The largest number of life years gained was obtained with 10-yearly colonoscopy and the lowest with CCE after FIT47+ screening. Both FIT15 and FIT47 screening, and 10-yearly colonoscopy were on the efficient frontier ( Fig. 2 ).

**Table:** **Table 4** Lifetime modeling outcomes (discounted at 3%) per 1000 simulated Dutch individuals for a situation without screening, fecal immunochemical test screening, colon capsule endoscopy (CCE) screening, CCE triage screening, and colonoscopy screening for three adherence scenarios.

	Life years	QALYs	Life years gained ^1^	QALYs gained ^1^	Costs, €	Costs, €/QALY
No screening	73 722	73 631	–	–	1 200 737	–
100% adherence						
Biennial FIT47	73 836	73 770	115	139	1 144 410	15.51
Biennial FIT15	73 861	73 801	140	171	1 154 074	15.64
CCE triage	73 839	73 774	118	143	1 206 442	16.35
CCE after FIT47+	73 804	73 730	83	99	1 258 541	17.07
CCE after FIT15+	73 816	73 745	95	114	1 294 641	17.56
Triennial CCE	73 818	73 748	97	118	1 700 084	23.05
10-yearly colonoscopy	73 908	73 855	187	224	1 705 087	23.09
Biennial CCE	73 876	73 817	155	186	3 178 861	43.06
75% adherence to FIT screening, CCE triage screening, and after FIT+; 25% adherence to CCE and colonoscopy screening						
Biennial FIT15	73 846	73 782	124	152	1 136 886	15.41
Biennial FIT47	73 821	73 751	99	120	1 138 769	15.44
CCE triage	73 825	73 756	103	125	1 187 947	16.11
CCE after FIT47+	73 792	73 715	71	85	1 238 537	16.80
CCE after FIT15+	73 805	73 731	83	100	1 265 668	17.17
10-yearly colonoscopy	73 792	73 717	71	86	1 286 205	17.45
Triennial CCE	73 769	73 688	47	57	1 426 027	19.35
Biennial CCE	73 793	73 717	71	86	1 674 192	22.71
75% adherence to FIT screening, CCE triage screening, and after FIT+; 50% adherence to CCE screening; 25% adherence to colonoscopy screening						
Biennial FIT15	73 846	73 782	124	152	1 136 886	15.41
Biennial FIT47	73 820	73 751	99	120	1 138 691	15.44
CCE triage	73 825	73 756	103	125	1 187 947	16.11
CCE after FIT47+	73 792	73 715	71	85	1 238 537	16.80
CCE after FIT15+	73 805	73 731	83	100	1 265 668	17.17
10-yearly colonoscopy	73 792	73 717	71	86	1 286 205	17.45
Triennial CCE	73 794	73 718	72	87	1 557 381	21.13
Biennial CCE	73 831	73 763	110	133	2 167 350	29.38
CCE, colon capsule endoscopy; CRC, colorectal cancer; FIT, fecal immunochemical test; µg Hb/g, microgram hemoglobin per gram feces; FIT15, FIT using a positivity cutoff of 15 µg Hb/g feces; FIT47, FIT using a positivity cutoff of 47 µg Hb/g feces; FIT+, positive FIT; FIT15+, positive FIT15; FIT47+, positive FIT47; QALY, quality-adjusted life year; ICER, incremental cost-effectiveness ratio. ^1^ Compared with no screening.						

**Fig. 3:**
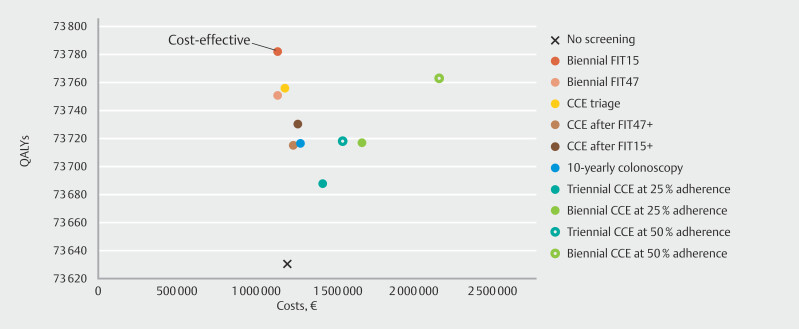
Life time costs and life years (discounted at 3%) per 1000 simulated Dutch individuals of all colorectal cancer screening strategies assuming two different participation rates for colon capsule endoscopy, and participation rates of 25% for colonoscopy and 75% for fecal immunochemical test, and a strategy without screening, with the efficient frontier connecting the economically efficient strategies. CCE, colon capsule endoscopy; FIT, fecal immunochemical test; FIT15, FIT using a positivity cutoff of 15 µg Hb/g feces; FIT47, FIT using a positivity cutoff of 47 µg Hb/g feces; QALY, quality-adjusted life year.

Using the WTP threshold of €20,000 per QALY gained, the optimal screening strategy at 100% adherence was 10-yearly colonoscopy screening (ICER, €10,311 per QALY gained). All CCE strategies were dominated, but CCE triage was close to the efficient frontier. At imperfect adherence, FIT15 was considered to be the optimal screening strategy ( Fig. 3 ).

### Sensitivity analysis

Our results were robust to changes in surveillance interval ( **Table 1s** , **Fig. 2s** ), participation rates of 50% and 75% ( **Table 2s** , **Table 3s** , **Fig. 3s** , **Fig. 4s** ) for all screening tests, and when incorporating the reviewing time into the unit costs of CCE ( **Table 4s** , **Fig. 5s** ). Under these assumptions, 10-yearly colonoscopy screening was the most cost-effective strategy. Biennial FIT15 screening was the most efficient strategy when assuming a participation rate of 25% for all screening tests ( **Table 5s** ; **Fig. 6s** ). Threshold analysis suggested that both biennial and triennial CCE screening could be cost effective with CCE unit costs of €42.94–65.28 and ≤€98.77, respectively. However, 10-yearly colonoscopy screening remained the optimal strategy using a WTP threshold of €20 000 per QALY gained. Only at a unit cost below €26.85, would biennial CCE screening be the optimal cost-effective screening strategy.

### Probabilistic sensitivity analysis

The probabilistic sensitivity analysis suggested that at the WTP threshold of €20 000 per QALY gained, 10-yearly colonoscopy screening was the most cost-effective strategy in all of 1000 considered cost values ( **Fig. 7s** ). None of the CCE screening strategies was cost effective in any of the 1000 values of the costs.

## Discussion

This study assessed the cost-effectiveness of several applications of CCE in CRC screening using the microsimulation model MISCAN-Colon. Our results suggest that CCE screening as a primary screening test is not cost effective compared with biennial FIT screening and 10-yearly colonoscopy in the Dutch population. Although CCE triage screening (offering CCE after a FIT-negative result between 15 and 47 µg Hb/g feces) was suggested to be not cost effective compared with FIT and colonoscopy screening, offering individuals CCE triage screening would probably be more cost effective than offering individuals a CCE after a positive FIT (FIT ≥47 µg Hb/g feces).

The lack of cost-effectiveness of CCE screening is mostly driven by the high cost of a single capsule compared with the low costs of a single FIT (€600 for a CCE compared with €22 for a FIT [ **Table 2** ]), as the reductions in CRC incidence and mortality are similar to those for biennial FIT screening owing to a relatively high sensitivity of FIT. A new screening test would have to have a very high performance in the detection of CRC and advanced adenoma to counterbalance the low cost of FIT. Threshold analysis suggested that reducing the unit cost of CCE to below €98.77 would result in cost-effective biennial CCE screening, and reducing the cost to €42.94–65.28 would make triennial CCE screening cost effective. However, CCE strategies remain suboptimal; unit costs would have to fall to below €26.85 for biennial CCE screening to become optimal. Moreover, although CCE has comparable sensitivities and costs as colonoscopy, specificity is much lower, resulting in a higher number of false-positive results and unnecessary follow-up colonoscopies; therefore, it is not cost effective compared with colonoscopy screening.

Although the cost-effectiveness of FIT and colonoscopy in CRC screening has been established extensively, the cost-effectiveness of CCE has been assessed less often, and never for the Dutch population, to the best of our knowledge. Cost-effectiveness studies comparing alternative strategies to FIT and colonoscopy screening have been conducted for the US population. One study used a mathematical Markov model to compare colonoscopy screening with CCE screening, concluding that CCE screening is not cost effective at similar participation rates but that CCE screening could be cost effective with higher participation rates for CCE than for colonoscopy, which is in line with the current study 26 . Another study using the MISCAN-Colon model in the US population also suggested that CCE is not cost effective compared with FIT and colonoscopy, even as an alternative test among other tests such as blood-based tests 19 .

A strength of this study is that we used a well-established microsimulation model to assess the cost-effectiveness of several screening strategies at the same time. However, our study has some limitations. First, we assumed 100% adherence in one of the adherence scenarios, which is an unrealistic participation rate; however, we aimed to compare different strategies fairly and to determine the effect on participating individuals. When assuming realistic participation rates based on invasiveness, CCE screening was still not (the most) cost-effective strategy. Even our sensitivity analyses suggested that only at a 25% participation rate for all screening modalities would biennial CCE screening be cost effective, while at 50% and 75%, biennial FIT screening and 10-yearly colonoscopy screening were more cost effective. Second, in the MISCAN-Colon model, localization is ordered according to the movement of the colonoscopy, which is from rectum to cecum, whereas the capsule moves from cecum to rectum. Therefore, we were not able to explicitly incorporate the reach of CCE into the model, which might result in an unrealistic performance of CCE. Third, we did not take the review time into account when modeling CCE screening. CCEs are reviewed by an endoscopy nurse, clinical nurse specialist, or nurse, with associated costs. This takes an average of 55 minutes 28 . Therefore, in practice, CCE requires healthcare capacity and costs in terms of staff hours. Our base case estimated costs of CCE screening are thereby an underestimation of the costs. However, including costs associated with reviewing does not change the conclusion about the cost-effectiveness of CCE screening, as shown in the sensitivity analysis. Artificial intelligence is expected to be a useful tool in the future for reviewing CCEs and would require significantly less capacity. Fourth, the 95%CIs around the costs are unknown and the chosen range is therefore uncertain. Nevertheless, applying the same methodology in determining the 95%CIs for the costs, CCE screening was not considered to be cost effective in any of the cost simulations used in the probabilistic sensitivity analysis.

CCE can also be used in triage to prioritize high-risk individuals for colonoscopy when colonoscopy capacity is temporarily restricted or as an alternative to colonoscopy. CCE triage was close to the efficient frontier and therefore close to being cost effective. Based on the concentration of hemoglobin in (FIT-positive) individuals, CCE can be provided to individuals testing below a certain cutoff to be directly referred for follow-up colonoscopy. However, offering FIT would probably be cost effective in times of restrictions in colonoscopy capacity because analysis of CCEs also requires capacity. CCE might be useful as an alternative to colonoscopy in individuals who are unwilling or unable to undergo colonoscopy. The accuracy of CCE is comparable to that of colonoscopy, and CCE can be performed at home, which makes it promising as an alternative to colonoscopy 2 .

CCE screening for CRC can be beneficial as it can be performed at home, similarly to FIT, it visualizes the gastrointestinal tract allowing detection of abnormalities, and its performance in the detection of CRC and advanced adenoma is comparable to that of colonoscopy. However, it still requires bowel preparation, similarly to colonoscopy, which should be performed adequately in order to visualize the complete gastrointestinal tract 21 . Although CCE has comparable performance to colonoscopy, its cost-effectiveness is dependent on the completion rate. The completion rate can be improved by adjusting and personalizing the bowel preparation for CCE based on age, stool pattern, history of abdominal surgery, body mass index, and fiber intake 28 .

In conclusion, CRC screening using CCE is not cost effective compared with FIT and colonoscopy screening. Only in the very unlikely situation that participation for all screening modalities drops to 25% would CCE be cost effective. However, it is unlikely that CCE screening has equal participation rates to FIT, as CCE is more invasive compared with FIT. Therefore, CCE screening should not be considered for primary CRC screening. Future studies should evaluate the cost-effectiveness of CCE in other settings, for example as an alternative strategy for those who are unwilling or cannot undergo a colonoscopy, and as a screening modality to detect multiple cancers during a single screening examination.

## Supplementary Material

Supplementary Material Zusatzmaterial
