# Supplementary material for: Cost-effectiveness of colon capsule endoscopy in colorectal cancer screening: a modeling study
Source: Endoscopy. 2025 Aug 1;57(10):1095–105. doi: 10.1055/a-2658-0960 (PMC12509280; doi:10.1055/a-2658-0960)

Supplementary Materials

Cost-effectiveness of colon capsule endoscopy in colorectal cancer screening: a modeling study

Lucie de Jonge, Esther Toes-Zoutendijk, Rosita van den Puttelaar, F. E. R. Vuik, Manon C. W. Spaander, Iris Lansdorp-Vogelaar

Tables

Table 1s *Lifetime modeling outcomes (discounted at 3%) using alternative surveillance intervals per 1000 simulated Dutch individuals for a situation without screening, fecal immunochemical test screening, colon capsule endoscopy screening, colon capsule endoscopy triage screening, colon capsule endoscopy after positive fecal immunochemical test, and colonoscopy screening.*

	FITs	CCEs	Colonoscopies	Complications	CRC cases	CRC deaths	LYs	QALYs	LYs gained[a]	QALYs gained[a]	Costs (€)	Costs (€)/QALY	ICER (€/QALYG)
No screening	-	-	-	-	79	36	73,722	73,631	-	-	1,200,737	-	Dominated
Biennial FIT47	8,899	-	619	0.08	53	21	73,839	73,774	118	143	1,175,513	15.93	Reference
Biennial FIT15	8,364	-	983	0.08	45	17	73,866	73,806	144	176	1,199,932	16.26	744
CCE triage	8,588	246	662	0.08	51	20	73,847	73,783	125	152	1,235,571	16.75	Dominated
CCE after FIT47+	9,129	280	374	0.08	61	25	73,809	73,735	87	105	1,276,344	17.31	Dominated
CCE after FIT15+	8,780	530	439	0.09	58	23	73,825	73,755	103	124	1,319,507	17.89	Dominated
10-yearly colonoscopy	-	-	3,343	0.08	27	10	73,913	73,860	191	229	1,789,332	24.23	10,998
Triennial CCE	-	3,102	546	0.10	55	22	73,842	73,776	120	145	2,017,939	27.35	Dominated
Biennial CCE	-	7,593	607	0.11	48	17	73,880	73,822	158	191	3,230,506	43.76	Dominated

Abbreviations: CRC, colorectal cancer; FIT, fecal immunochemical test; µg Hb/g, microgram Hemoglobin per gram; FIT15, FIT using a positivity cutoff of 15 µg Hb/g feces; FIT47, FIT using a positivity cutoff of 47 µg Hb/g feces; FIT15+, positive FIT15; FIT47+, positive FIT47; CCE, colon capsule endoscopy; LYs, life years; QALYs, quality-adjusted life years; QALYG, QALY gained; ICER, incremental cost-effectiveness ratio.

a. (QA)LYs gained compared to a situation without screening.

***Table 2s** Lifetime modeling outcomes (discounted at 3%) at 50% participation rate per 1000 simulated Dutch individuals for a situation without screening, fecal immunochemical test screening, colon capsule endoscopy screening, colon capsule endoscopy triage screening, colon capsule endoscopy after positive fecal immunochemical test and colonoscopy screening.*

	FITs	CCEs	Colonoscopies	Complications	CRC cases	CRC deaths	LYs	QALYs	LYs gained[a]	QALYs gained[a]	Costs (€)	Costs (€)/QALY	ICER (€/QALYG)
No screening	-	-	-	-	79	36	73,722	73,631	-	-	1,200,737	-	Dominated
Biennial FIT15	4,252	-	565	0.08	54	22	73,823	73,755	102	125	1,127,543	15.29	Reference
Biennial FIT47	4,501	-	350	0.07	61	25	73,800	73,726	78	95	1,138,200	15.44	Dominated
CCE triage	4,342	136	387	0.07	59	25	73,804	73,731	82	100	1,175,175	15.94	Dominated
CCE after FIT47+	4,585	159	224	0.05	67	29	73,776	73,696	54	65	1,221,684	16.58	Dominated
CCE after FIT15+	4,406	287	280	0.07	64	27	73,788	73,711	66	80	1,238,427	16.80	Dominated
10-yearly colonoscopy	-	-	1,727	0.07	44	18	73,844	73,779	123	149	1,405,427	19.05	11,626
Triennial CCE	-	1,419	336	0.07	63	27	73,794	73,718	72	87	1,557,381	21.13	Dominated
Biennial CCE	-	3,783	439	0.10	56	22	73,831	73,763	110	133	2,167,350	29.38	Dominated

Abbreviations: CRC, colorectal cancer; FIT, fecal immunochemical test; µg Hb/g, microgram Hemoglobin per gram; FIT15, FIT using a positivity cutoff of 15 µg Hb/g feces; FIT47, FIT using a positivity cutoff of 47 µg Hb/g feces; FIT15+, positive FIT15; FIT47+, positive FIT47; CCE, colon capsule endoscopy; LYs, life years; QALYs, quality-adjusted life years; QALYG, QALY gained; ICER, incremental cost-effectiveness ratio.

a. (QA)LYs gained compared to a situation without screening.

Thieme

Table 3s *Lifetime modeling outcomes (discounted at 3%) at 75% participation rate per 1000 simulated Dutch individuals for a situation without screening, fecal immunochemical test screening, colon capsule endoscopy screening, colon capsule endoscopy triage screening, colon capsule endoscopy after positive fecal immunochemical test and colonoscopy screening.*

	FITs	CCEs	Colonoscopies	Complications	CRC cases	CRC deaths	LYs	QALYs	LYs gained[a]	QALYs gained[a]	Costs (€)	Costs (€)/QALY	ICER (€/QALYG)
No screening	-	-	-	-	79	36	73,722	73,631	-	-	1,200,737	-	Dominated
Biennial FIT15	6,120	-	710	0.08	49	20	73,846	73,782	124	152	1,136,886	15.41	Reference
Biennial FIT47	6,608	-	443	0.07	56	23	73,820	73,751	99	120	1,138,691	15.44	Dominated
CCE triage	6,285	186	474	0.08	55	22	73,825	73,756	103	125	1,187,947	16.11	Dominated
CCE after FIT47+	6,764	209	277	0.06	64	27	73,792	73,715	71	85	1,238,537	16.80	Dominated
CCE after FIT15+	6,401	383	330	0.07	61	25	73,805	73,731	83	100	1,265,668	17.17	Dominated
10-yearly colonoscopy	-	-	2,446	0.08	34	13	73,882	73,824	160	193	1,548,193	20.97	9,883
Triennial CCE	-	1,747	390	0.09	60	25	73,809	73,736	87	106	1,645,057	22.31	Dominated
Biennial CCE	-	5,624	496	0.11	52	19	73,857	73,794	136	164	2,669,711	36.18	Dominated

Abbreviations: CRC, colorectal cancer; FIT, fecal immunochemical test; µg Hb/g, microgram Hemoglobin per gram; FIT15, FIT using a positivity cutoff of 15 µg Hb/g feces; FIT47, FIT using a positivity cutoff of 47 µg Hb/g feces; FIT15+, positive FIT15; FIT47+, positive FIT47; CCE, colon capsule endoscopy; LYs, life years; QALYs, quality-adjusted life years; QALYG, QALY gained; ICER, incremental cost-effectiveness ratio.

a. (QA)LYs gained compared to a situation without screening.

Table 4s *Lifetime modeling outcomes (discounted at 3%) including cost for reviewing time of CCE per 1000 simulated Dutch individuals for a situation without screening, fecal immunochemical test screening, colon capsule endoscopy screening, colon capsule endoscopy triage screening, colon capsule endoscopy after positive fecal immunochemical test and colonoscopy screening.*

	FITs	CCEs	Colonoscopies	Complications	CRC cases	CRC deaths	LYs	QALYs	LYs gained[a]	QALY gained[a]	Costs (€)	Costs (€)/QALY	ICER (€/QALYG)
No screening	-	-	-	-	79	36	73,722	73,631	-	-	1,200,737	-	-
Biennial FIT47	8,659	-	516	0.07	53	21	73,836	73,770	115	139	1,144,410	15.51	Reference
Biennial FIT15	7,875	-	829	0.09	46	18	73,861	73,801	140	171	1,154,074	15.64	308
CCE triage	8,124	231	541	0.08	53	21	73,839	73,774	118	143	1,223,809	16.59	Dominated
CCE after FIT47+	8,895	253	312	0.08	62	25	73,804	73,730	83	99	1,277,346	17.32	Dominated
CCE after FIT15+	8,296	468	362	0.07	59	24	73,816	73,745	95	114	1.329.578	18,03	Dominated
10-yearly colonoscopy	-	-	3,089	0.07	29	11	73,908	73,855	187	224	1,705,087	23.09	10,311
Triennial CCE	-	1,939	423	0.09	59	24	73,818	73,748	97	118	1,855,963	25.17	Dominated
Biennial CCE	-	7,459	530	0.11	49	17	73,876	73,817	155	186	3,732,932	50.57	Dominated

Abbreviations: CRC, colorectal cancer; FIT, fecal immunochemical test; µg Hb/g, microgram Hemoglobin per gram; FIT15, FIT using a positivity cutoff of 15 µg Hb/g feces; FIT47, FIT using a positivity cutoff of 47 µg Hb/g feces; FIT15+, positive FIT15; FIT47+, positive FIT47; CCE, colon capsule endoscopy; LYs, life years; QALYs, quality-adjuste life years; QALYG, QALY gained; ICER, incremental cost-effectiveness ratio.

a. (QA)LYs gained compared to a situation without screening.

Table 5s *Lifetime modeling outcomes (discounted at 3%) at 25% participation rate per 1000 simulated Dutch individuals for a situation without screening, fecal immunochemical test screening, colon capsule endoscopy screening, colon capsule endoscopy triage screening, colon capsule endoscopy after positive fecal immunochemical test and colonoscopy screening.*

	FITs	CCEs	Colonoscopies	Complications	CRC cases	CRC deaths	LYs	QALYs	LYs gained[a]	QALYs gained[a]	Costs (€)	Costs (€)/QALY	ICER (€/QALYG)
No screening	-	-	-	-	79	36	73,722	73,631	-	-	1,200,737	16.31	Dominated
Biennial FIT15	2,239	-	361	0.06	62	26	73,789	73,713	67	82	1,135,425	15.40	Reference
Biennial FIT47	2,315	-	219	0.05	67	29	73,770	73,690	48	59	1,154,164	15.66	Dominated
CCE triage	2,269	77	252	0.05	66	29	73,774	73,694	52	64	1,174,944	15.94	Dominated
CCE after FIT47+	2,343	95	141	0.04	72	31	73,754	73,670	32	39	1,207,366	16.39	Dominated
CCE after FIT15+	2,291	168	188	0.05	69	30	73,764	73,682	42	51	1,213,892	16.47	Dominated
10-yearly colonoscopy	-	-	918	0.04	58	25	73,792	73,717	71	86	1,286,205	17.45	42,210
Triennial CCE	-	911	232	0.06	68	30	73,769	73,688	47	57	1,426,027	19.35	Dominated
Biennial CCE	-	1,925	320	0.07	63	26	73,793	73,717	71	86	1,674,192	22.71	134,716

Abbreviations: CRC, colorectal cancer; FIT, fecal immunochemical test; µg Hb/g, microgram Hemoglobin per gram; FIT15, FIT using a positivity cutoff of 15 µg Hb/g feces; FIT47, FIT using a positivity cutoff of 47 µg Hb/g feces; FIT15+, positive FIT15; FIT47+, positive FIT47; CCE, colon capsule endoscopy; LYs, life years; QALYs, quality-adjusted life years; QALYG, QALY gained; ICER, incremental cost-effectiveness ratio.

a. (QA)LYs gained compared to a situation without screening.

without screening with the efficient frontier connecting the economically efficient strategies.

Fig. 1s Screening pathway used in the MISCAN-Colon microsimulation model for colon capsule endoscopy triage screening for those with fecal immunochemical test result between 15-47 microgram Hemoglobin per gram feces.
Abbreviations: FIT, fecal immunochemical test; Hb, Hemoglobin; fHb, fecal Hb.

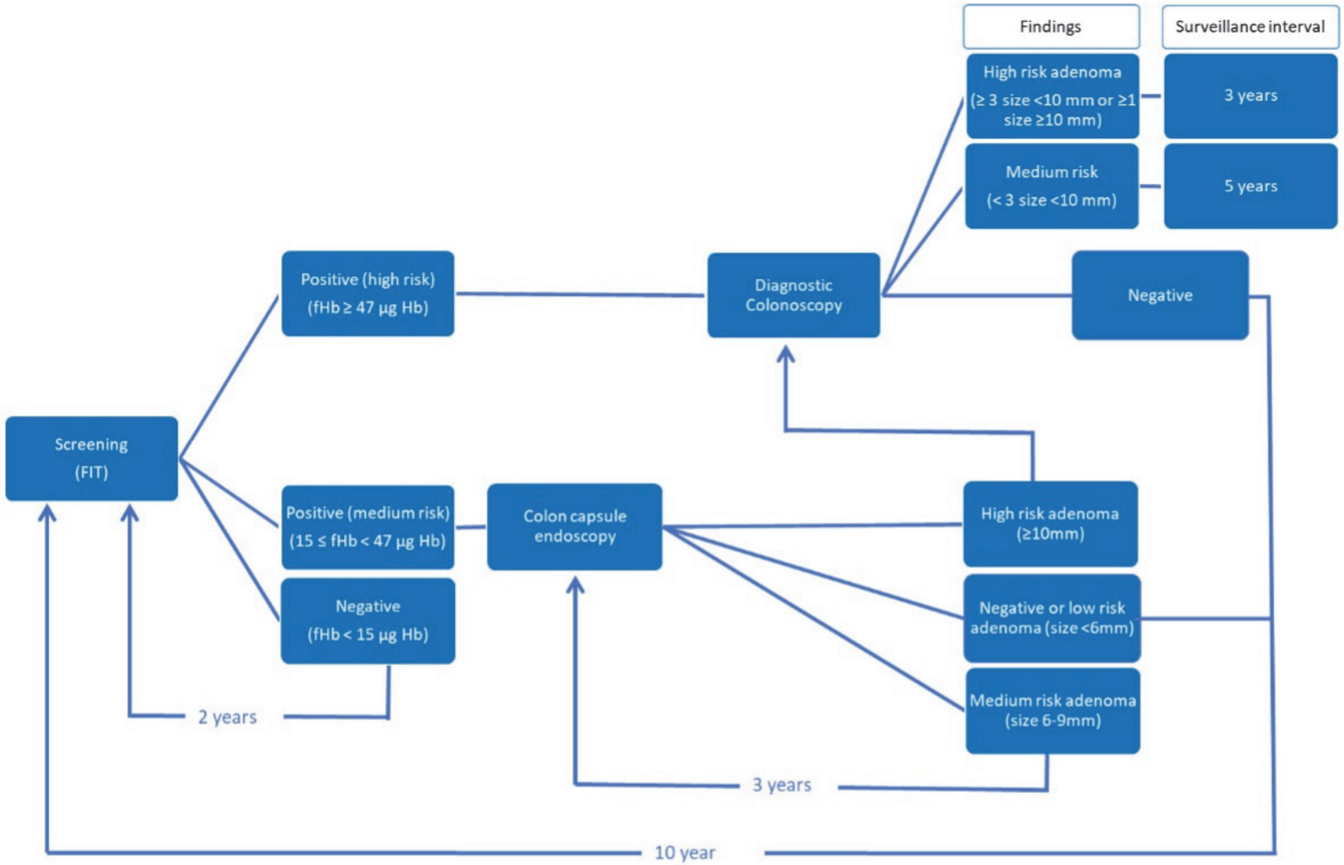

Thieme

Fig. 2s *Lifetime costs and life years (discounted at 3%) per 1000 simulated Dutch individuals of all colorectal cancer screening strategies and a strategy without screening using alternative surveillance intervals, with the efficient frontier connecting the economically efficient strategies.*
Abbreviations: CRC, colorectal cancer; FIT, fecal immunochemical test; µg Hb/g, microgram Hemoglobin per gram; FIT15, FIT using a positivity cutoff of 15 µg Hb/g feces; FIT47, FIT using a positivity cutoff of 47 µg Hb/g feces; FIT15+, positive FIT15; FIT47+, positive FIT47; CCE, colon capsule endoscopy; QALYs, quality-adjusted life years.

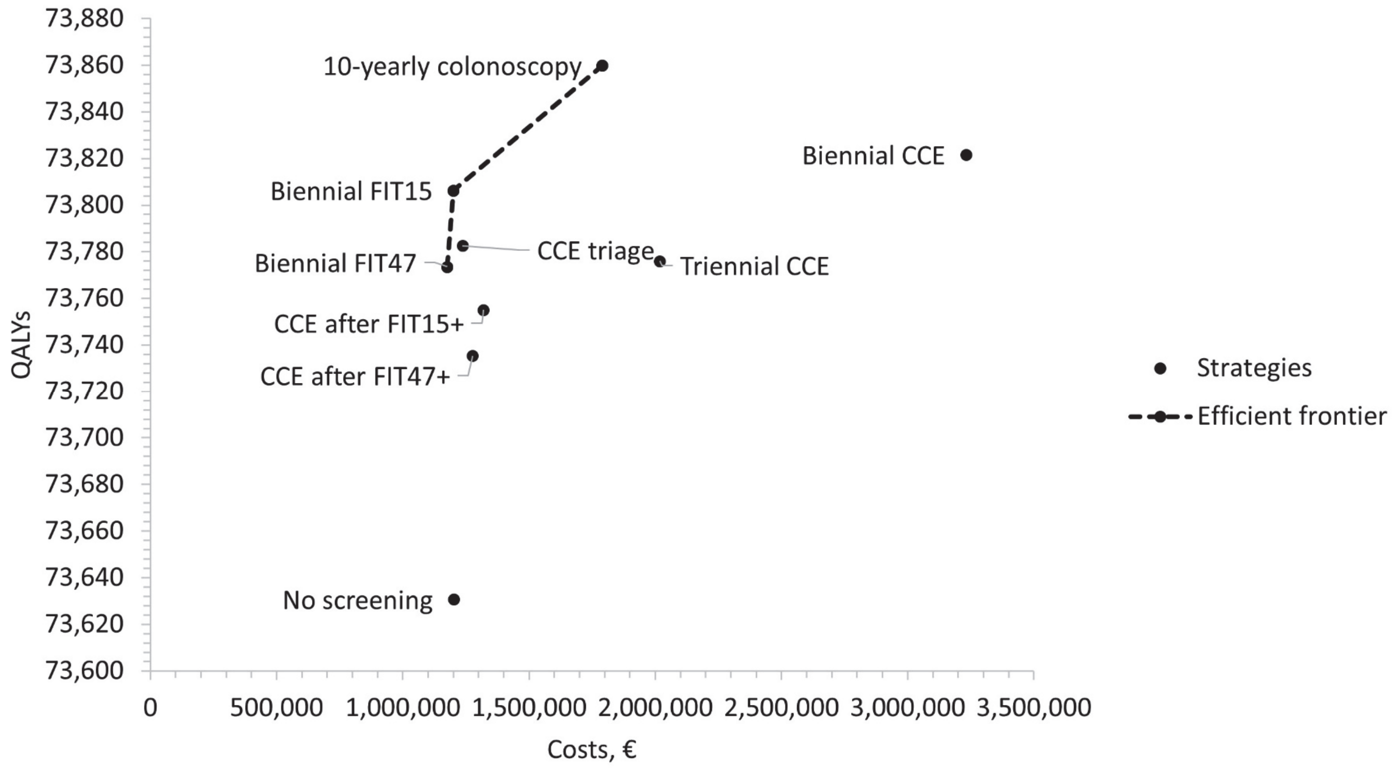

Fig. 3s *Lifetime costs and life years (discounted at 3%) per 1000 simulated Dutch individuals of all colorectal cancer screening strategies with assumed participation rate of 50% and a strategy without screening with the efficient frontier connecting the economically efficient strategies.*
Abbreviations: CRC, colorectal cancer; FIT, fecal immunochemical test; µg Hb/g, microgram Hemoglobin per gram; FIT15, FIT using a positivity cutoff of 15 µg Hb/g feces; FIT47, FIT using a positivity cutoff of 47 µg Hb/g feces; FIT15+, positive FIT15; FIT47+, positive FIT47; CCE, colon capsule endoscopy; QALYs, quality-adjusted life years.

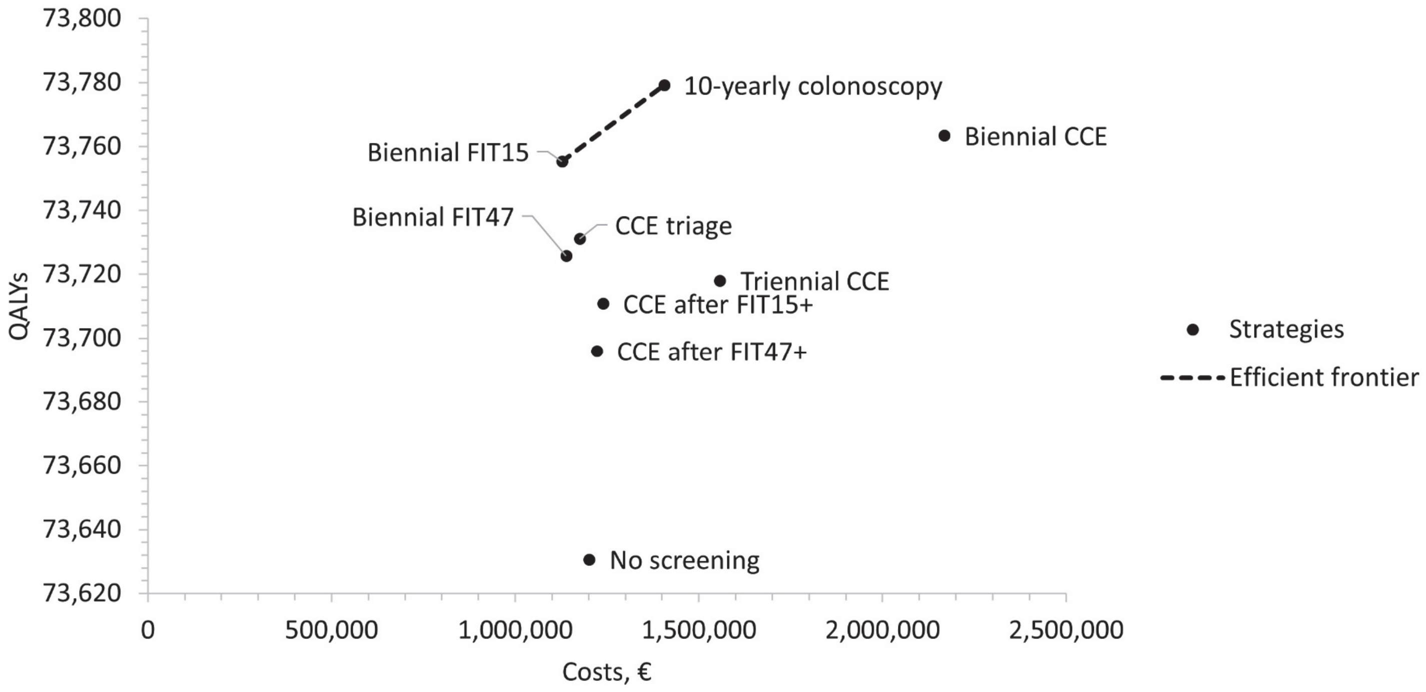

Fig. 4s *Lifetime costs and life years (discounted at 3%) per 1000 simulated Dutch individuals of all colorectal cancer screening strategies with assumed participation rate of 75% and a strategy without screening with the efficient frontier connecting the economically efficient strategies.*
Abbreviations: CRC, colorectal cancer; FIT, fecal immunochemical test; µg Hb/g, microgram Hemoglobin per gram; FIT15, FIT using a positivity cutoff of 15 µg Hb/g feces; FIT47, FIT using a positivity cutoff of 47 µg Hb/g feces; FIT15+, positive FIT15; FIT47+, positive FIT47; CCE, colon capsule endoscopy; QALYs, quality-adjusted life years.

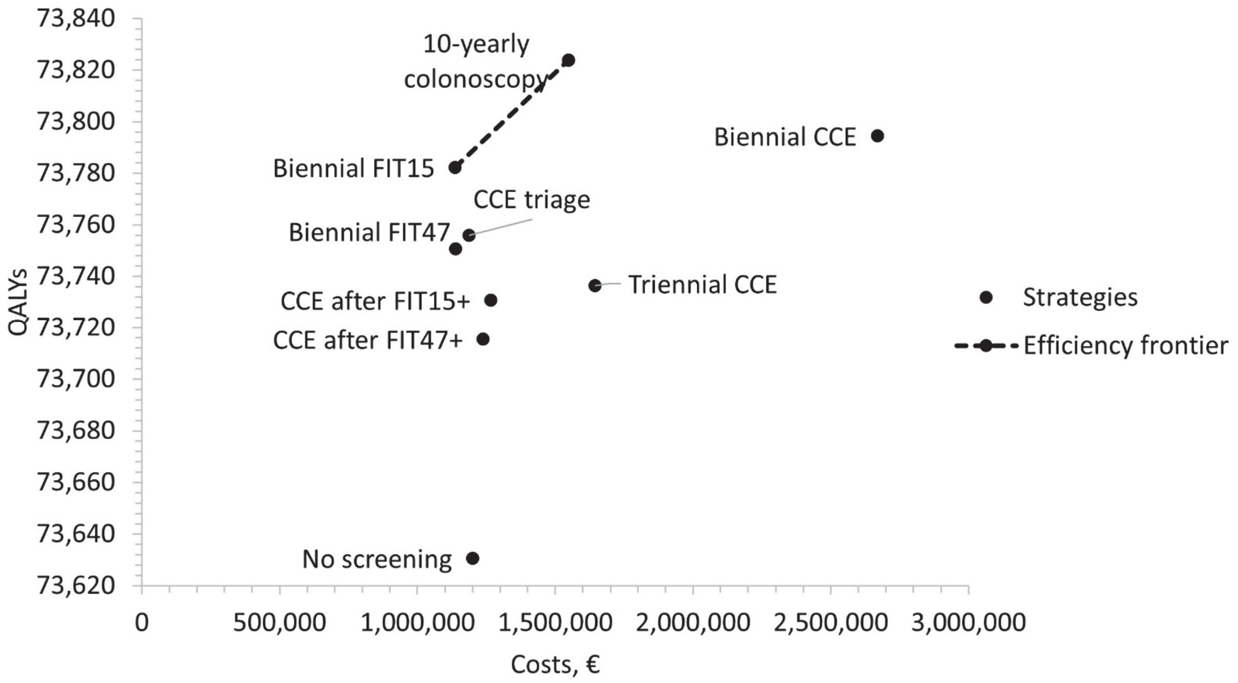

***Fig. 5s** Lifetime costs and life years (discounted at 3%) per 1000 simulated Dutch individuals of all colorectal cancer screening strategies including costs of the reviewing time of CCE and a strategy without screening with the efficient frontier connecting the economically efficient strategies.*
Abbreviations: CRC, colorectal cancer; FIT, fecal immunochemical test; µg Hb/g, microgram Hemoglobin per gram; FIT15, FIT using a positivity cutoff of 15 µg Hb/g feces; FIT47, FIT using a positivity cutoff of 47 µg Hb/g feces; FIT15+, positive FIT15; FIT47+, positive FIT47; CCE, colon capsule endoscopy; QALYs, quality-adjusted life years.

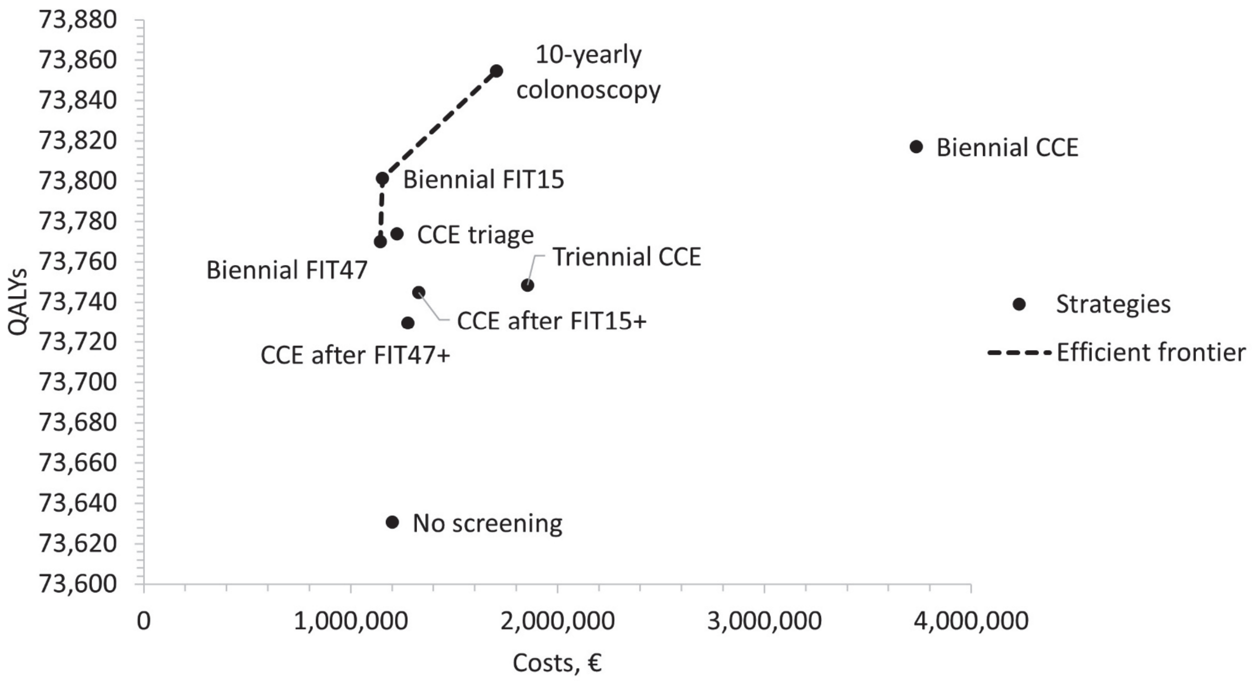

Fig. 6s Lifetime costs and life years (discounted at 3%) per 1000 simulated Dutch individuals of all colorectal cancer screening strategies with assumed participation rate of 25% and a strategy without screening with the efficient frontier connecting the economically efficient strategies.

Abbreviations: CRC, colorectal cancer; FIT, fecal immunochemical test; µg Hb/g, microgram Hemoglobin per gram; FIT15, FIT using a positivity cutoff of 15 µg Hb/g feces; FIT47, FIT using a positivity cutoff of 47 µg Hb/g feces; FIT15+, positive FIT15; FIT47+, positive FIT47; CCE, colon capsule endoscopy; QALYs, quality-adjusted life years.

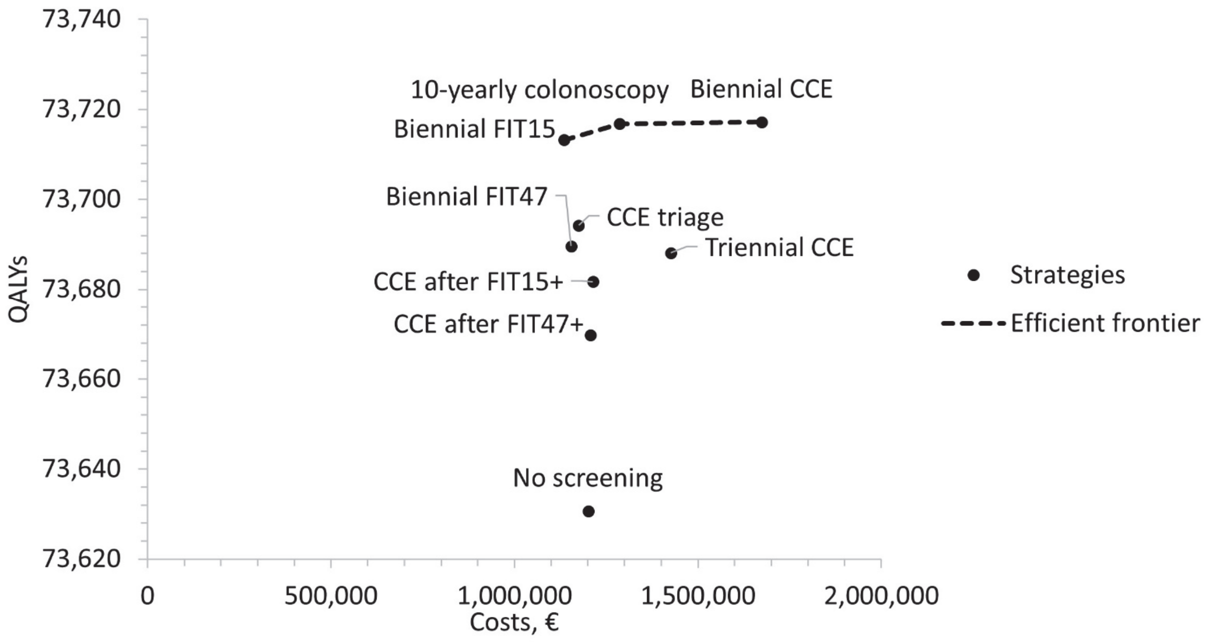

Fig. 7s Cost-effectiveness acceptability curve of the probabilistic sensitivity analysis.
Abbreviations: CCE, colon capsule endoscopy; FIT, fecal immunochemical test; FIT15/47, FIT positivity cut-off of 15/47 microgram per gram feces; QALY, Quality adjusted life year.

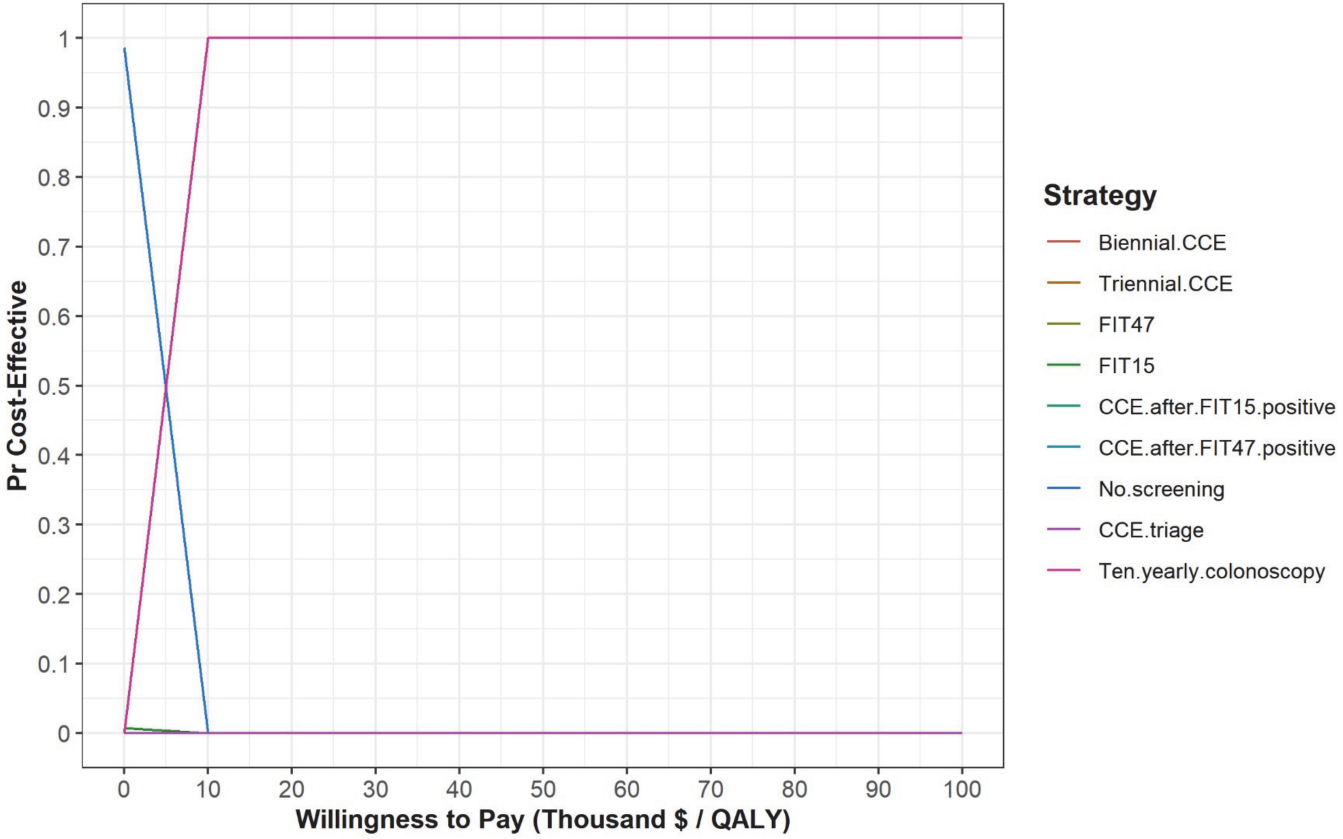

Supplement: Supplementary file 1 — Supplementary Material [file 10-1055-a-2658-0960_26582034.pdf]
